# Machine Learning Application: A Bibliometric Analysis From a Half-Century of Research on Stroke

**DOI:** 10.7759/cureus.44142

**Published:** 2023-08-26

**Authors:** Che Muhammad Nur Hidayat Che Nawi, Suhaily Mohd Hairon, Wan Nur Nafisah Wan Yahya, Wan Asyraf Wan Zaidi, Mohd Rohaizat Hassan, Kamarul Imran Musa

**Affiliations:** 1 Department of Community Medicine, School of Medical Sciences, Universiti Sains Malaysia, Kubang Kerian, MYS; 2 Department of Internal Medicine/ Neurology, Universiti Kebangsaan Malaysia Medical Centre, Kuala Lumpur, MYS; 3 Department of Community Health, Faculty of Medicine, National University of Malaysia, Kuala Lumpur, MYS

**Keywords:** stroke research, neural networks, algorithm, deep learning, bibliometric analyses, stroke, machine learning

## Abstract

The quick advancement of digital technology through artificial intelligence has made it possible to deploy machine learning to predict stroke outcomes. Our aim is to examine the trend of machine learning applications in stroke-related research over the past 50 years.

We used search terms stroke and machine learning to search for English versions of original and review articles and conference proceedings published over the past 50 years in Scopus and Web of Science databases. The Biblioshiny web application was utilized for the analysis. The trend of publication and prominent authors and journals were analyzed and identified. The collaborative network between countries was mapped, and a thematic map was used to monitor the authors' trending keywords. In total, 10,535 publications authored by 44,990 authors from 2,212 sources were retrieved. Two distinct clusters of collaborative network nodes were observed, with the United States serving as a connecting node. Three terms - deep learning, algorithms, and neural networks - are observed in the early stages of the emerging theme. Overall, international research collaborations, the establishment of global research initiatives, the development of computational science, and the availability of big data have facilitated the pervasive use of machine learning techniques in stroke research.

## Introduction and background

Globally, stroke is the main cause of death and disability. The absolute number of incident strokes increased by 70.0% (67.0-73.0), prevalent strokes increased by 85.0% (83.0-88.0), deaths from stroke increased by 43.0% (31.0-55.0), and disability-adjusted life years (DALYs) attributable to stroke increased by 32.0% (22.0-42.0) between 1990 and 2019. [1]. Besides, 63% and 80% of stroke cases happen among people less than 70 years old and have a low to moderate cardiovascular disease absolute risk, respectively. Furthermore, nearly 90% of the global stroke deaths and disability combined reside in low-to-middle-income countries [2].

Nowadays, stroke research with structured data applying machine learning (ML) algorithms for outcome prediction has gained more popularity [3]. The complexity of a condition and the availability of routinely collected datasets like stroke may lend themselves well to the application of ML methods, which can incorporate a large number of variables and observations into a single predictive framework. There were a number of studies that incorporated various machine-learning algorithms to predict stroke outcomes in various global populations [4-6]. Mortality, functional outcome, duration of hospital stay, and neurologic deterioration were the post-stroke outcomes most frequently predicted. In addition, artificial neural network (ANN), support vector machine (SVM), decision tree (DT), and random forest (RF) were the most popular machine learning algorithms [3].

Both stroke and machine learning have been the subject of extensive research in the past. However, no investigation has been conducted into how these two distinct topics have been studied together. As the focal point of scientific evaluation, it is necessary to evaluate the growth, advancement, and impact of global research on these overlapping issues. Bibliometric analysis is a newly developed method for revealing patterns in published research. It evaluates an exhaustive literature search that demonstrates advancements within the field. Bibliometric analysis is an effective method for obtaining quantitative data on particular subjects [7]. Additionally, recognizing influential researchers and publications, countries to take into account for collaboration, and popular keywords on the application of ML in stroke research provide relevant references to the stakeholders working on stroke research to address current issues like a high stroke burden and research limitations.

In light of this, the purpose of this study was to examine the trend of machine learning applications in stroke research. Specifically, we examined its trend over the past 50 years and identified distinctive collaborative network clusters and emerging themes.

## Review

Methodology

Source of Data and Eligibility Criteria

The Scopus and Web of Science databases were selected for this bibliometric analysis. Scopus and Web of Science were databased by Elsevier and Clarivate, respectively. Only original English-language articles, reviews, and conference proceedings published before August 1, 2022, were eligible. Other publications, including editorials and letters, were excluded.

Search Strategy

**Figure 1:**
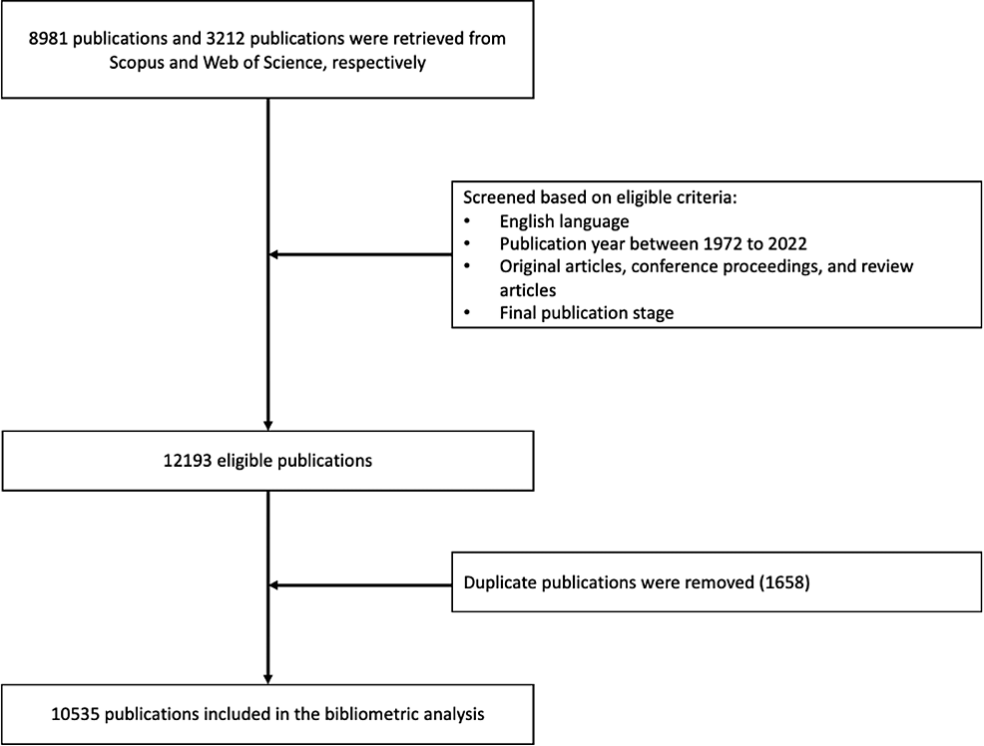
The flowchart of the research process for selecting publications in databases for the study

The terms "stroke" and "machine learning" were searched in Scopus and Web of Science on August 1, 2022, for the article title, abstract, and keywords using the advanced search option in the respective databases and an appropriate combination of Boolean search operators. The flowchart of our investigation is depicted in Figure 1.

Appendix A contains an in-depth search strategy for the study.

Bibliometric Analyses

The BibTex format was used to retrieve and obtain the metadata consisting of all the primary information and characteristics of the publications included in the analysis. Data management and bibliometric analyses were performed utilizing the Bibliometrix package and Biblioshiny web applications under the R programming language (version 4.2.1, RStudio: Integrated Development for R. RStudio, PBC, Boston, MA, USA) [8,9]. The BibTex format was used to retrieve and obtain all the primary information and characteristics of the publications used in the analysis. The development of the publication trend spanned 50 years.

Based on the total number of publications and Hirsch (H)-indexed citations, we identified the top 10 most influential authors and sources. The total number of publications is defined as the number of publications produced within 50 years. Meanwhile, the H-index is computed by counting the number of publications for which an author has been cited at least that many times by other authors. The highest number of publications and H-indexed citations will be in the top rank and followed by others. The influence of the top 10 sources was then tabulated in order to compare the total number of citations and H-indexed citations.

The publication by country was visualized, and a collaboration network was established between the top 43 countries that published the most articles during the time period. The frequency of the authors' keywords was plotted against the years to illustrate the dominant topics during the specified time period. Four themes were used to construct the 'thematic map'. They were (i) motor themes, (ii) niche themes, (iii) emerging or declining themes, and (iv) transversal and fundamental themes. On the map, co-occurrence networks were used to identify concentrations of keyword plus, which are keywords assigned by journals after extracting words and phrases from the titles of cited articles.

Results

**Figure 2:**
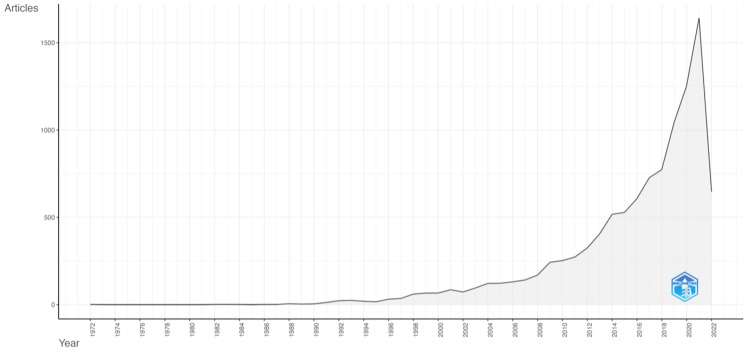
Annual scientific publications on stroke and machine learning from 1972 to 2022

Based on our study criteria, 8,981 and 3,212 publications were retrieved from Scopus and Web of Science, respectively. After screening for duplicate publications, we finally determined that 10,535 publications were eligible for analysis. The publications were from 2,212 sources and consisted of 9,653 original articles (91.6%), 216 review papers (2.1%), and 666 conference proceedings (6.3%). The annual publication growth rate is 13.8%, with average citations of 28.7 for each publication, as depicted in Figure 2.

Authors and Journals

Within the 50-year period, 44,990 authors were involved in publications regarding the application of ML to stroke research. Most publications (99%) were co-authored, with an average of 9.03 authors per publication. Table 1 lists the top 10 authors who published the most works over a 50-year span.

**Table 1:** Top 10 authors who have published research work on stroke and machine learning research over five decades

Rank	Element	Field of interest	Institution	Country	Total number of publications	H-indexed publications	G-indexed publications	M-indexed publications	Total citations	Start of publication year
1	Yanzhong Wang	Stroke epidemiology	King’s College London,	United Kingdom	387	40	91	1.667	9695	2000
2	Kennedy R. Lees	Stroke, Internal medicine, cardiology	University of Glasgow	United Kingdom	62	34	62	1.214	5413	1996
3	Ralph L. Sacco	Neurology, public health	University of Miami	USA	77	34	69	0.971	4813	1989
4	Diederik Dippel	Neurology	Erasmus University Rotterdam	Netherlands	93	31	83	1.192	6903	1998
5	Stephen Davis	Neurology	University of Melbourne	Australia	48	30	48	1.364	3907	2002
6	Andrew Demchuk	Neurology	University of Calgary	Canada	67	30	65	1.2	4286	1999
7	Mitchell S. V. Elkind	Neurology	Columbia University	USA	67	30	54	1.25	3040	2000
8	Joon-Tae Kim	Neuroradiology	Chonnam National University	South Korea	150	30	51	1.364	3145	2002
9	Carlos Molina	Neurology	Hospital Universitari Vall d'Hebron	Spain	43	30	43	1.304	4654	2001
10	Chun-Fu Chen	Neurology	Shandong University	China	97	29	44	1.706	2145	2007

The author Yanzhong Wang from King’s College London, United Kingdom, was the most impactful author (387 publications, 3.67%), followed by Joon-Tae Kim from Chonnam National University, South Korea. Although the author Kennedy R. Lees produced less than half of Joon-Tae Kim’s publications, he was in the second rank with 34 H-indexed publications, followed by Ralph L. Sacco (34 H-indexed publications) and Diederik Dippel (31 H-indexed publications).

The top 10 most relevant journals publishing research on the application of ML in stroke research are listed in Table 2.

**Table 2:** The top 10 journals with the highest number of publications, journal impact, and the start year of publication on the topic

Rank	Element	Total number of publications	H-indexed publications	G-indexed publications	M-indexed publications	Total citations	Start of publication year
1	Stroke	1073	145	214	3.919	76449	1987
2	Neurology	152	51	85	1.545	7947	1991
3	Circulation	59	48	59	1.171	17734	1983
4	PLOS One	332	41	61	2.412	6648	2007
5	Journal of Vascular Surgery	130	37	62	1.028	4698	1988
6	Cerebrovascular Diseases	132	35	49	1.094	3436	1992
7	Journal of Thoracic and Cardiovascular Surgery	59	34	59	1.063	5130	1992
8	Journal of Neurology, Neurosurgery and Psychiatry	45	31	45	1	2944	1993
9	Archives of Neurology	33	29	33	0.935	3330	1993
10	American Journal of Neuroradiology	92	28	48	1.4	2666	2004

Table 2 includes the name of the journal, total number of publications, citation impact parameters, and start publication years. Since 1987, the Stroke Journal has been the most productive and impactful source, with over one thousand publications (145 H-indexed publications) published. As a comparison, the Neurology journal has the second highest number of H-indexed publications (51) followed by the Circulation journal (48 H-indexed publications) and the PLOS One journal (41 H-indexed publications). Although the start of the publication year for PLOS One and the American Journal of Neuroradiology journals is later than the other journals, these journals were ranked among the tenth most impactful journals with 332 publications (41 H-indexed publications) and 92 publications (28 H-indexed publications) within a shorter period. All 10 journals that are involved in publications regarding the application of ML to stroke research are specifically related to cardiovascular and cerebrovascular topics, except for PLOS One, which is an open-access and multidisciplinary journal.

Countries and Collaboration Network

Overall, authors from 107 countries have contributed to the publications on the application of ML to stroke research. The United States (2,484 publications, Single Country Production (SCP) of 2,439, Multiple Country Production (MCP) of 55) dominated the within and interstate production of stroke and machine learning research publications, followed by China (1,888 publications, SCP of 1,840, MCP of 48), the United Kingdom (517 publications, SCP of 505, MCP of 12), and Korea (476 publications, SCP of 467, MCP of 9). Table 3 provides a summary of the top 10 countries, along with their total publications, annual publication rate, single-country publications, multi-country publications, and their proportions.

**Table 3:** The top 10 countries contributing publications relating to stroke and machine learning research SCP: single country production; MCP: multiple country production

Rank	Country	Number of publications	Frequency	SCP	MCP	MCP_SCP ratio
1	United States of America	2494	0.237	2439	55	0.022
2	China	1888	0.179	1840	48	0.025
3	United Kingdom	517	0.049	505	12	0.023
4	Korea	476	0.045	467	9	0.019
5	Japan	456	0.043	451	5	0.011
6	Germany	414	0.039	390	24	0.058
7	Netherlands	316	0.03	313	3	0.009
8	Canada	299	0.028	277	22	0.074
9	Spain	251	0.024	236	15	0.06
10	Australia	218	0.021	208	10	0.046

**Figure 3:**
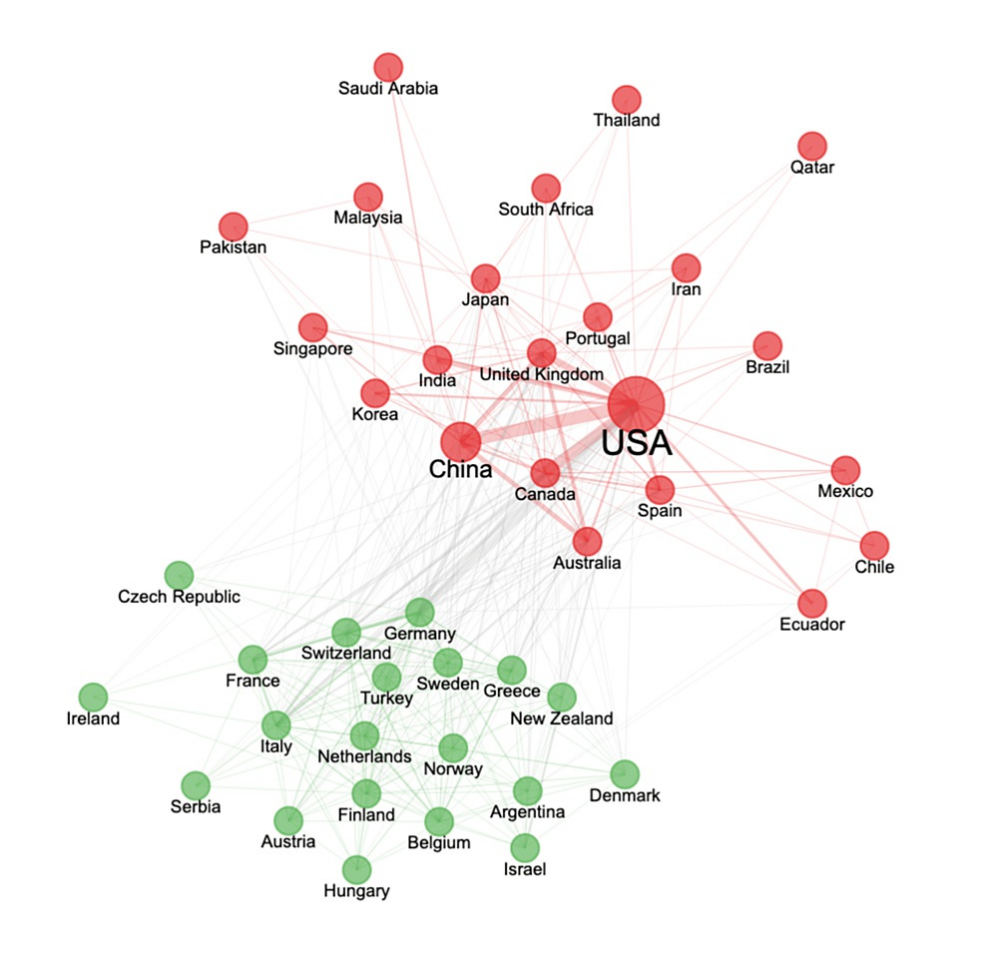
A collaborative network of countries producing research on stroke and machine learning USA: United States of America

In the top 43 countries, two separate clusters of collaborative networks may be seen, as shown in Figure 3.

The bubble represents countries, while the connection lines between the bubbles represent their collaboration. The larger bubble is related to more publications, and the thicker line is related to their research collaboration activities. The first cluster (red bubbles) is made up of American and Asian countries, with the USA acting as the central node. In contrast, the second cluster (green bubbles) consists primarily of European countries, with Germany acting as the central node.

Trending Keywords and Thematic Map

**Figure 4:**
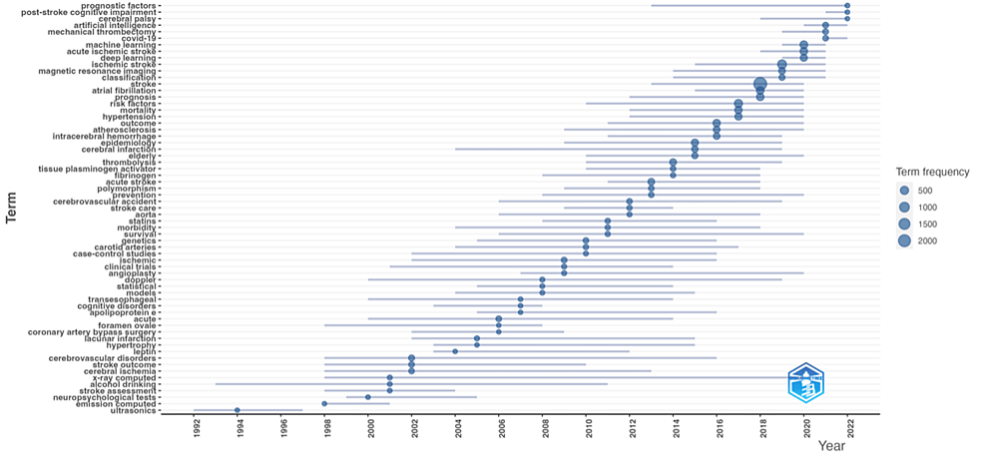
Trending topics and keywords for stroke and machine learning research from 1972 to 2022. The line shows the keyword's occurrence, while the bubble size shows its frequency throughout the year. COVID-19: coronavirus disease 2019

Figure 4 shows the 63 trending author keywords that were found over the past 50 years of using ML in stroke research.

In the top keywords, we found four patterns. Firstly, the change in the stroke diagnostic imaging keyword from x-ray computed (1998-2020), which was gradually replaced by magnetic resonance imaging (2014-2021). Secondly, in terms of post-stroke assessment and outcome measures, the keywords have changed gradually from general (neuropsychological tests, stroke outcome, cognitive disorder, and stroke care within 1998-2014) to specific assessment and outcome measures of stroke (stroke prognosis, prognostic factors, COVID-19, and post-stroke cognitive disorder within 2012-2022). Thirdly, we noticed a shift in the types of risk factors under study. Among the risk factors, alcohol and genetics were trending from 1998 to 2016, which gradually shifted to the elderly, atherosclerosis, hypertension, and atrial fibrillation from 2009 to 2020. Finally, in terms of statistical analysis of stroke research, the keywords shifted from model-based regression analysis in 2003 to model-free analysis in 2019.

**Figure 5:**
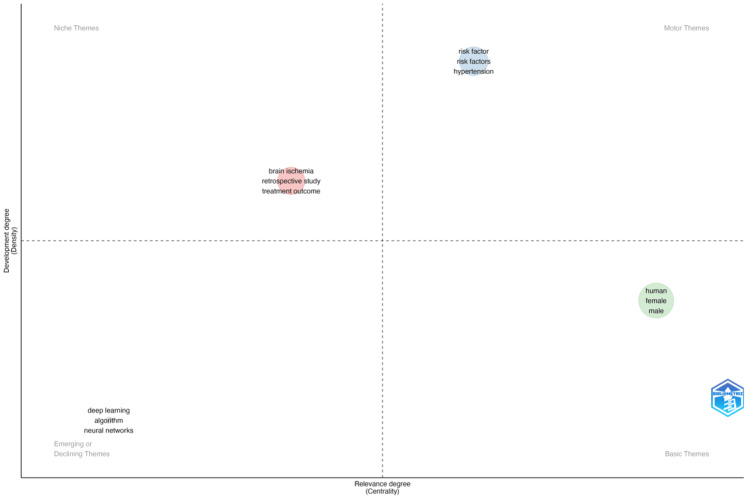
Thematic map of authors' keywords on stroke and machine learning research

Thematic maps represent keyword clusters as bubbles based on Callon's centrality (x-axis) and density (y-axis) ranks. The bubble size reflects the cluster's word frequency. Centrality measures the relationship between network clusters in the same graph. It indicates a theme's importance to research advancement. The density measures cluster network internal strength and subject development. The three keywords (a) deep learning, b) algorithms, and c) neural networks are observed in the early development stages of emerging themes (Figure 5).

Besides, stroke classification, research study method, and treatment outcome are observed in niche themes. Meanwhile, modifiable and non-modifiable stroke risk factors are observed in motor and basic themes, respectively.

Discussion

Bibliometric analysis using publication data from 1972-2022 shows that the publication on the application of ML in stroke research experienced a slow rise at first and a rapid rise thereafter. However, there is a notable decline in 2022, as the publication data were collected through August 2022. The steep decline does not accurately reflect the publication trend of stroke and machine learning in 2022. Besides, notable authors and institutions reside in the United Kingdom, Australia, and the United States. Furthermore, the United States served as the connecting node between two distinct clusters of collaborative networks. The terms deep learning, algorithm, and neural networks were classified in the early phase of an emerging theme.

The establishment of the World Stroke Organization (WSO) in 2006 has had a significant impact on stroke research [10]. The WSO was established to bring together professionals, organizations, and policymakers worldwide in the fight against stroke, aiming to reduce its global impact through prevention and improved care. Its membership encompasses 92 countries, fostering collaboration and the exchange of knowledge. The WSO contributes by increasing stroke awareness, providing evidence-based guidelines, promoting research, and advocating globally for improved stroke care and rehabilitation services [10-12]. Additionally, the availability of structured stroke data from multicenter stroke registries that are accessible for research, the development of computing capacity, and the availability of machine learning models all contribute to the research activity in this area [3].

All 10 prominent authors in stroke and machine learning research hail from diverse regions, including the United Kingdom, the Netherlands, Spain, China, South Korea, Canada, and the USA. Among them, Yanzhong Wang stands out as an exception, being a professor of statistics at King’s College, London, focusing on stroke epidemiology. His expertise lies in clinical prediction models and the application of machine learning in stroke research [3,13]. Notably, in comparison to his clinician counterparts, Yanzhong Wang boasts a significantly higher number of publications, H-indexed citations, and other influential parameters. This data underscores the necessity for multidisciplinary collaboration in the realm of advanced machine learning stroke modeling, calling for expertise from statistics, clinical practice, and computer sciences.

The three most active countries in producing publications related to the application of machine learning in stroke research are the USA, China, and the United Kingdom. These countries collectively contribute to nearly half of the global stroke publications. This higher publication volume is attributed to the increased degree of collaboration among them. Notable examples illustrating this principle are initiatives such as Stroke Therapy Academic Industry Roundtable (STAIR) and Stem Cell Therapies as an Emerging Paradigm in Stroke (STEPS), which play a pivotal role in guiding and supporting translational stroke research [14]. These initiatives exemplify effective collaborative networks between stroke experts, particularly from the USA and other nations within the first cluster (depicted by red bubbles).

Furthermore, the progress of stroke research is furthered by the support provided by the Leducq Foundation, which fosters laboratories to pursue mechanisms and targets related to stroke and cardiovascular diseases [15]. The Leducq Foundation has facilitated numerous research collaborations encompassing countries from both the first and second clusters, including the USA, Canada, the United Kingdom, Germany, France, Belgium, Sweden, and Austria.

Moreover, Safe Implementation of Treatments in Stroke (SITS) and the Virtual International Stroke Trials Archive (VISTA) stand as pivotal organizations that offer platforms and databases for the international analysis and advancement of stroke therapies [16,17]. Datasets from SITS and VISTA have been harnessed in a variety of studies to predict stroke outcomes through machine learning techniques [5,18,19]. Ultimately, all endeavors in advancing stroke research converge on the overarching goal of saving lives and enriching the quality of life for those affected by stroke.

From 1972 to 2022, the trend of publications supported the idea that researchers' interest in applying ML to stroke research has grown. This can be depicted by the change of author keywords from model-based to model-free analysis and the emergence of keywords related to machine learning in Figures 4-5 of our analyses. The model-based analysis uses standard regression methods such as logistic regression and Cox proportional hazard regression. Meanwhile, the model-free analysis uses machine learning algorithms such as decision trees, artificial neural networks, and random survival forests. The improvement of computational capacity and the availability of model-free algorithms further catalyze the use of machine learning in stroke research. To predict the outcomes of a stroke, machine learning employs vast, frequently collected datasets to generate accurate, individualized prognoses. These ML models-artificial neural networks (ANN), Nave Bayes, support vector machines (SVM), decision trees (DT), and random forests (RF)-have been utilized to predict stroke mortality [3].

There are a few limitations in our analysis. We cannot retrieve the actual number of publications on the ML application on stroke because some publications were written in languages other than English. Moreover, financial limitations and other various reasons might hinder the submission of manuscripts to the journals. These will result in not all publications being available in the Web of Science and Scopus databases. Thus, future studies may compare our findings to data from more databases with a wider diversity of language styles.

## Conclusions

In summary, the bibliometric analysis of the application of ML in stroke research from 1972 to 2022 reveals a significant growth in the application of machine learning (ML) in this field. The United Kingdom, Australia, and the United States are prominent contributors to this field, and international collaborations, supported by initiatives and organizations, have played a crucial role in advancing stroke research. The trend indicates a shift towards ML analysis and the emergence of ML-related keywords, reflecting the increasing interest and utilization of ML algorithms in the multifaceted management of stroke care. Besides, computational advancements and the availability of big data have facilitated the widespread application of machine learning techniques in stroke research. These findings highlight the potential of ML to enhance stroke prevention, treatment, and patient care. Greater emphasis should be placed on augmenting research endeavors to stimulate increased publication output, particularly within the Southeast Asian region.

## Appendices

Appendix A

Search strategy in Scopus and Web of Science

**Table 4:** Database search terms for Scopus and Web of Science

Databases	Search terms
Scopus	TITLE-ABS ( "Strokes" OR "Cerebrovascular Accident" OR "Cerebrovascular Accidents" OR "CVA (Cerebrovascular Accident)" OR "CVAs (Cerebrovascular Accident)" OR "Cerebrovascular Apoplexy" OR "Apoplexy, Cerebrovascular" OR "Vascular Accident, Brain" OR "Brain Vascular Accident" OR "Brain Vascular Accidents" OR "Vascular Accidents, Brain" OR "Cerebrovascular Stroke" OR "Cerebrovascular Strokes" OR "Stroke, Cerebrovascular" OR "Strokes, Cerebrovascular" OR "Cerebral Stroke" OR "Cerebral Strokes" OR "Stroke, Cerebral" OR "Strokes, Cerebral" OR "Stroke, Acute" OR "Acute Stroke" OR "Acute Strokes" OR "Strokes, Acute" OR "Cerebrovascular Accident, Acute" OR "Acute Cerebrovascular Accident" OR "Acute Cerebrovascular Accidents" OR "Cerebrovascular Accidents, Acute" ) AND TITLE-ABS ( "machine learning" OR "machine learnings" OR "deep learning" OR "Decision tree" OR "Classification tree" OR "Tree-based" OR "Support vector" OR "Transfer learning" OR "neural network" OR "neural networks" OR "Logistic regression" OR "discriminant analysis" OR "k-nearest neighbor" OR "k-nearest neighbour" OR "nearest neighbor" OR "nearest neighbour" OR bayes OR "random forest" ) AND ( LIMIT-TO ( OA , "all" ) ) AND ( LIMIT-TO ( DOCTYPE , "ar" ) OR LIMIT-TO ( DOCTYPE , "cp" ) OR LIMIT-TO ( DOCTYPE , "re" ) ) (TITLE-ABS=((cerebrovascular accident $ OR apoplexy OR brain vascular accident $ OR cva$ OR cerebral stroke $ OR Cerebral Infarction OR cerebrovascular stroke $ OR stroke$) OR ( ischemi* AND (stroke $ OR post Stroke OR poststroke)) OR ( (transient AND ischemi*) OR tia$ ) OR ( ( h$emorrhag* OR (carotid AND artery) OR (carotid AND stenos*) OR (carotid AND ulcer$)) AND (Stroke$ OR poststroke OR post stroke)))) AND (TITLE-ABS=(machine learning OR Artificial Intelligence OR Naive Bayes OR bayesian learning OR Neural network$ OR Natural language processing OR support vector machine$ OR random forest$ OR boosting OR deep learning OR machine intelligence OR computational intelligence OR computer reasoning))
Web of Science	#1: (AB=("Strokes" OR "Cerebrovascular Accident" OR "Cerebrovascular Accidents" OR "CVA (Cerebrovascular Accident)" OR "CVAs (Cerebrovascular Accident)" OR “Cerebrovascular Apoplexy” OR “Apoplexy, Cerebrovascular” OR "Vascular Accident, Brain" OR "Brain Vascular Accident" OR "Brain Vascular Accidents" OR "Vascular Accidents, Brain" OR "Cerebrovascular Stroke" OR "Cerebrovascular Strokes" OR "Stroke, Cerebrovascular" OR "Strokes, Cerebrovascular" OR "Cerebral Stroke" OR "Cerebral Strokes" OR "Stroke, Cerebral" OR "Strokes, Cerebral" OR "Stroke, Acute" OR "Acute Stroke" OR "Acute Strokes" OR "Strokes, Acute" OR "Cerebrovascular Accident, Acute" OR "Acute Cerebrovascular Accident" OR "Acute Cerebrovascular Accidents" OR "Cerebrovascular Accidents, Acute")) AND AB=("machine learning" OR "machine learnings" OR "deep learning" OR "Decision tree" OR "Classification tree" OR "Tree-based" OR "Support vector" OR "Transfer learning" OR "neural network" OR "neural networks" OR "Logistic regression" OR "discriminant analysis" OR "k-nearest neighbor" OR "k-nearest neighbour" OR "nearest neighbor" OR "nearest neighbour" OR bayes OR "random forest") #2: (TI=("Strokes" OR "Cerebrovascular Accident" OR "Cerebrovascular Accidents" OR "CVA (Cerebrovascular Accident)" OR "CVAs (Cerebrovascular Accident)" OR “Cerebrovascular Apoplexy” OR “Apoplexy, Cerebrovascular” OR "Vascular Accident, Brain" OR "Brain Vascular Accident" OR "Brain Vascular Accidents" OR "Vascular Accidents, Brain" OR "Cerebrovascular Stroke" OR "Cerebrovascular Strokes" OR "Stroke, Cerebrovascular" OR "Strokes, Cerebrovascular" OR "Cerebral Stroke" OR "Cerebral Strokes" OR "Stroke, Cerebral" OR "Strokes, Cerebral" OR "Stroke, Acute" OR "Acute Stroke" OR "Acute Strokes" OR "Strokes, Acute" OR "Cerebrovascular Accident, Acute" OR "Acute Cerebrovascular Accident" OR "Acute Cerebrovascular Accidents" OR "Cerebrovascular Accidents, Acute")) AND TI=("machine learning" OR "machine learnings" OR "deep learning" OR "Decision tree" OR "Classification tree" OR "Tree-based" OR "Support vector" OR "Transfer learning" OR "neural network" OR "neural networks" OR "Logistic regression" OR "discriminant analysis" OR "k-nearest neighbor" OR "k-nearest neighbour" OR "nearest neighbor" OR "nearest neighbour" OR bayes OR "random forest") #1 OR #2
